# Supplementary material for: Ageing as risk factor for tinnitus and its complex interplay with hearing loss—evidence from online and NHANES data
Source: BMC Med. 2023 Aug 2;21:283. doi: 10.1186/s12916-023-02998-1 (PMC10394883; doi:10.1186/s12916-023-02998-1)
Supplement: Supplementary file 1 — Additional file 1. More details of the model and input variables. Table S1. Spearman’s Rank Correlation between the online hearing test and pure-tone audiometry over four frequencies and split by ear (n=93). Table S2. Summary of variables in the NHANES dataset, split into a tinnitus group and a non-tinnitus group. Fig. S1. Correlation matrix of the relevant variables in the NHANES dataset. Table S3. Summary of the simple and complex model of the NHANES data. [file 12916_2023_2998_MOESM1_ESM.docx]

Additional file 1

Table S1. Spearman’s Rank Correlation between the online hearing test and pure-tone audiometry over four frequencies and split by ear (n=93).

|  | 1000 Hz | 2000 Hz | 4000 Hz | 6000 Hz |
| --- | --- | --- | --- | --- |
| Left Ear | .602 | .653 | .832 | .824 |
| Right Ear | .411 | .618 | .747 | .776 |

Table S2. Summary of variables in the NHANES dataset, split into a tinnitus group and a non-tinnitus group.

|  | Tinnitus | No Tinnitus |
| --- | --- | --- |
| Hearing Loss | Mean=22.00 dB (sd=16.22) | Mean=11.22 dB (sd=10.36) |
| Age | Mean= 46.56 years (sd=22.62) | Mean= 36.12 years (sd=18.92) |
| Age Group | 343 younger  674 older | 7645 younger  7346 older |
| Sex | 561 male  456 female | 7403 male  7588 female |

Fig. S1. Correlation matrix of the relevant variables in the NHANES dataset.

*
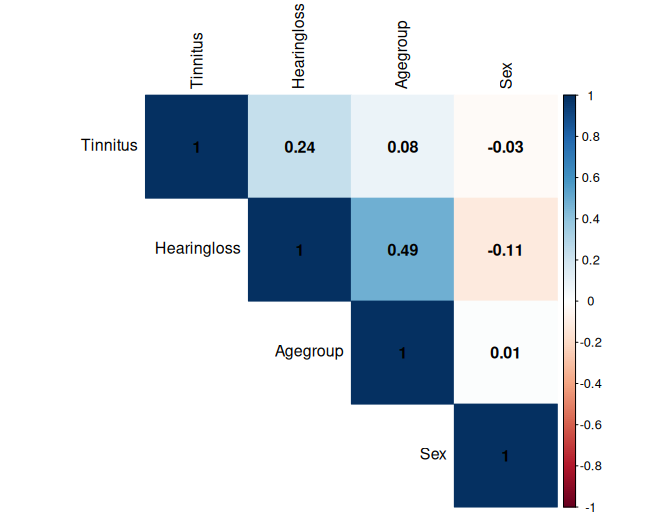
*

Table S3. Summary of the simple and complex model of the NHANES data.

$$tinnitus\sim agegroup+hearingloss$$

|  | Exponent | Std Error | z Value | p-Value |
| --- | --- | --- | --- | --- |
| Intercept | -3.597 | 0.075 | -48.2 | <.001 *** |
| Agegroup | 0.058 | 0.080 | 0.72 | .469 |
| Hearing Loss | 0.057 | 0.002 | 23.0 | <.001 *** |

$$tinnitus\sim agegroup*hearingloss$$

|  | Exponent | Std Error | z Value | p-Value |
| --- | --- | --- | --- | --- |
| Intercept | -3.701 | 0.081 | -45.7 | <.001 *** |
| Agegroup | 0.352 | 0.111 | 3.18 | .001 ** |
| Hearing Loss | 0.061 | 0.003 | 22.5 | <.001 *** |
| Agegroup:Hearing Loss | -0.027 | 0.007 | -3.81 | <.001 *** |

Significance codes: <.001: *** , <.01: ** , <.05: * , <.1: .
